# Framework for Quantification of the Dynamics of Root Colonization by *Pseudomonas fluorescens* Isolate SBW25

**DOI:** 10.3389/fmicb.2020.585443

**Published:** 2020-09-25

**Authors:** Daire Carroll, Nicola Holden, Miriam L. Gifford, Lionel X. Dupuy

**Affiliations:** ^1^Ecological Sciences, The James Hutton Institute, Dundee, United Kingdom; ^2^School of Life Sciences, University of Warwick, Coventry, United Kingdom; ^3^Northern Faculty, Scotland’s Rural College, Aberdeen, United Kingdom; ^4^Warwick Integrative Synthetic Biology Centre, University of Warwick, Coventry, United Kingdom; ^5^Neiker, Department of Conservation of Natural Resources, Derio, Spain; ^6^IKERBASQUE, Basque Foundation for Science, Bilbao, Spain

**Keywords:** rhizosphere, microbiome, bacterial dynamics, attachment, colonization, root surface, *Pseudomonas fluorescens*

## Abstract

Colonization of the root surface, or rhizoplane, is one of the first steps for soil-borne bacteria to become established in the plant microbiome. However, the relative contributions of processes, such as bacterial attachment and proliferation is not well characterized, and this limits our ability to comprehend the complex dynamics of microbial communities in the rhizosphere. The work presented here addresses this knowledge gap. A model system was developed to acquire quantitative data on the colonization process of lettuce (*Lactuca sativa* L. cultivar. All Year Round) roots by *Pseudomonas fluorescens* isolate SBW25. A theoretical framework is proposed to calculate attachment rate and quantify the relative contribution of bacterial attachment to colonization. This allows the assessment of attachment rates on the root surface beyond the short time period during which it can be quantified experimentally. All techniques proposed are generic and similar analyses could be applied to study various combinations of plants and bacteria, or to assess competition between species. In the future this could allow for selection of microbial traits that improve early colonization and maintenance of targeted isolates in cropping systems, with potential applications for the development of biological fertilizers.

## Introduction

The region of soil under direct influence of a plant root is termed the rhizosphere. The rhizosphere hosts a complex microbiome, distinct from both the bulk soil and other plant associated environments (Lundberg et al., 2012). Biological interactions taking place on the surface of the root shape microbial diversity in the rhizosphere. Successful colonization of the root surface, or rhizoplane, is often the first step toward entering the plant microbiome for soil-borne bacteria, including pathogens (Walker et al., 2004). High levels of microbial competition are observed at or near the surface of the root because bacteria seek plant-derived nutrients and space in which to establish themselves (Reinhold-Hurek et al., 2015). Colonization of the rhizoplane is an important step prior to internalization and translocation of bacteria within plant tissue (Berggren et al., 2005). The root surface is the point at which many plant growth promoting, and pathogen suppressing, bacteria are established and act to influence the plant (Köhl et al., 2019; Shinde et al., 2019). The rhizoplane is also susceptible to colonization by human pathogens (Wright et al., 2017).

Understanding the process of root surface colonization is challenging. Soil is a heterogeneous environment and enables very diverse forms of biological activity (Hinsinger et al., 2009; Kuzyakov and Blagodatskaya, 2015). Plants and microorganisms secrete a broad range of chemical compounds which can impact bacterial growth and alter their physiology (Dennis et al., 2010; Yu et al., 2019). Plants are known to recruit certain bacteria while suppressing others through immune responses (Chowdhury et al., 2015; Feng et al., 2019). Differences in community structure are observed at different stages of root development, or depending on root anatomy (Humphris et al., 2005; Schmidt et al., 2018). Bacteria associated with roots are also impacted by temporal variations in root exudation (Kuzyakov and Blagodatskaya, 2015).

Colonization of the rhizoplane requires complex and often very specific mechanisms that develop in a progressive manner. First, bacteria must be able to detect the presence of a root. Subsequently, bacteria must be able to move toward the root and then establish themselves in a location from which plant-derived nutrients are accessible. The chemotactic response of soilborne bacteria to plant derived signaling molecules has been well documented (Pliego et al., 2008; Feng et al., 2019). Mobility of microbes in soils and toward roots has been demonstrated in a many soil-borne bacteria. Bacterial motility and physical soil properties determine the ability of bacteria to approach the root. For example, soil moisture has been found to be the main factor effecting the movement of *Pseudomonas fluorescens* toward wheat roots (Bashan, 1986). As a result of this movement, higher microbial diversity is observed in the soil directly surrounding the root relative to that of the bulk soil (Robbins et al., 2018). Bacterial numbers on the rhizoplane can increase in two ways; (i) recruitment from the surrounding medium resulting in attachment and/or (ii) proliferation of established bacteria on the root. Bacteria can form weak reversible bonds, then strong permanent attachments to the root surface (Rossez et al., 2014). Root growth leads to the dilution of bacterial density on the rhizoplane and, eventually, displacement of bacterial colonies from sites of heavy exudation (Dupuy and Silk, 2016). It is likely that bacterial mobility on the rhizoplane contributes to maintenance of colonization at sites of exudation, but this has not been well studied.

Bacteria vary significantly in their ability to move, attach, and proliferate in the rhizosphere. In soil, dynamic interactions take place between the root and microbes which can either compete or cooperate during colonization (Lareen et al., 2016). The rhizosphere microbiome structure emerges as a result of these interactions. Recent developments in genomics, sequencing and bioinformatics have revealed the taxonomic diversity and positioning of bacteria within the rhizosphere. The specificity of certain taxonomic groups to host plants and environmental conditions has also been investigated through microbiome analysis (Dawson et al., 2017; Lucaciu et al., 2019). Bioinformatics approaches are increasingly focusing on extracting information on community functional traits (Fitzpatrick et al., 2018). Unfortunately, top-down molecular approaches lack the ability to identify factors that contribute to the maintenance of bacteria in the rhizosphere. To date, however, these approaches have only had a limited impact on agriculture and our ability to manipulate the plant microbiome (Gopal and Gupta, 2016). This may be due to a lack of understanding of the mechanisms through which bacteria are recruited and maintained on the root surface.

To address this knowledge gap, the work presented here proposes a mathematical framework to dissect the factors contributing to maintenance and recruitment on the rhizoplane. This framework links the relative contribution of attachment and proliferation on the rhizoplane to the overall colonization rate of the root. A model system was developed to acquire quantitative data on the colonization process of lettuce roots by *Pseudomonas fluorescens* isolate SBW25, an isolate with well characterized interaction with plants (Jackson et al., 2005). Through a series of colonization experiments we determined the key parameters that need to be measured in order to characterize microbial colonization. A theoretical framework is proposed to calculate attachment rate and quantify the relative contribution of recruitment to colonization. We have developed the techniques here to be able to be applied to study other combinations of plant and bacterial isolates, alone or in competition, thus the work has broad impact and value.

## Materials and Methods

### Plant Growth and Microcosm Set Up

**FIGURE 1:**
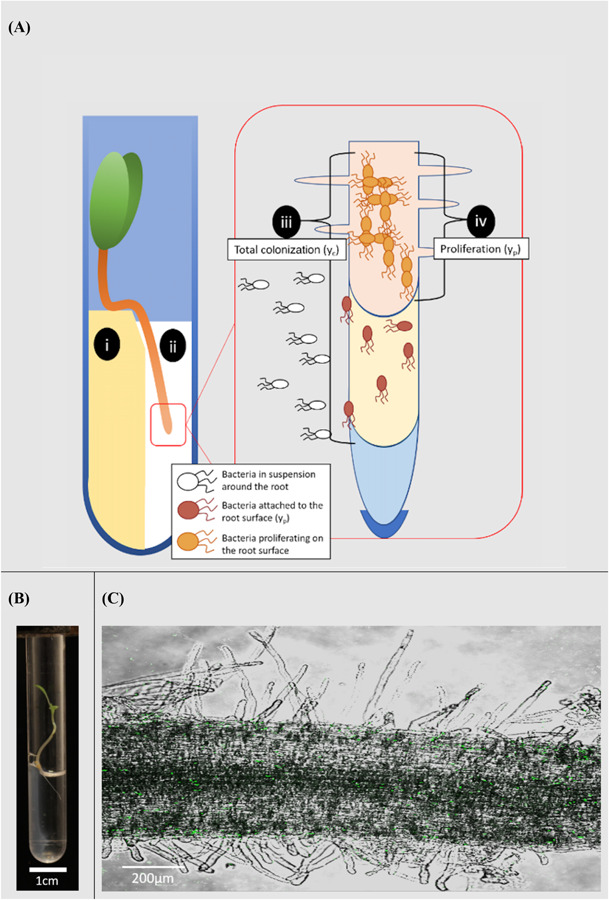
Microcosm system for the study of rhizoplane colonization. **(A)** Diagram of the microcosm system; (i) plants are grown on a water agar slope. (ii) A bacterial suspension is introduced to a level no higher than the hypocotyl. The system allows bacterial movement along the root and quantification of the colonization process; (iii) total colonization (y_c_) was the result of both attachment to and proliferation on the root surface. (iv) Proliferation on the root surface (y_p_) was quantified in the absence of attachment. **(B)** Microcosm chamber containing a growing lettuce seedling. **(C)** A confocal image of *Pseudomonas fluorescens* SBW25 E1433 (shown in green) superimposed over a brightfield image of a lettuce root.

Lettuce (*Lactuca sativa* L. cultivar. All Year Round) seeds were obtained from Sutton Seeds, United Kingdom. Prior to germination, seeds were surface sterilized by soaking in a solution of 2% w/v calcium hypochlorite (Sigma Aldrich 12116) for 15 min. They were subsequently washed in sterile distilled water. Seeds were then plated on 1.5% water agar. Plates were sealed and covered with foil. They were then incubated at 21°C for 3 days. Sterile microcosms were set up in 75 mm round bottom culture tubes (VWR 211-0046). 1.5% water agar (1 ml) was melted and pipetted into culture tubes. Tubes were set on their sides to allow agar to form a slope and a well in which microbial suspensions in liquid solution could interact with the root. Once agar had set, a small section was removed to form a platform (Figures 1A,B).

Each microcosm contained 1 ml of 0.5 × concentration Murashige and Skoog (MS) media without sucrose (Sigma Aldrich M5524). Light was prevented from reaching the roots by covering the bottom part of the tube with tape. Following germination, individual plants were transferred from plates to microcosms. They were placed on the water agar platform, with their root tip in the well (Figure 1A). Microcosms were then sealed, using a plastic lid, and placed in a growth-cabinet. Growth conditions were 21°C with 16 h of light at 60 μmol m^–2^ s^–1^. Plants were grown for 3 days before further treatment.

To assess the efficiency of surface sterilization, 20 seeds were sterilized as described above, imprints were made by placing sterilized seeds on Lysogeny Broth (LB) (Sigma Aldrich L9234) agar for ∼30 s, followed by incubation at either 18 or 27°C for 24 h.

### Bacterial Isolates and Bacterial Transformation

*Pseudomonas fluorescens* isolate SBW25 (genome accession AM181176.4) (Rainey and Bailey, 1996) was transformed with a fluorescent marker plasmid E1433 pGFP (Heeb et al., 2000). The resulting isolate was referred to as *P. fluorescens* SBW25 E1433. This was used as the model isolate for all subsequent experiments. The E1433 pGFP plasmid conferred tetracycline resistance to transformed bacteria. The E1433 pGFP plasmids was transformed into competent *P. fluorescens* isolate SBW25 by electroporation. Transformed bacteria were isolated by plating on LB agar containing tetracycline (25 μg ml^–1^). To test the stability of the plasmid, *P. fluorescens* SBW25 E1433 was grown in liquid LB and RD-MOPS (Neidhardt et al., 1974) in the absence of tetracycline. Cultures were incubated at 27°C, with shaking (200 rpm). Every 24 h, for 7 days, fresh 1:100 subcultures were prepared, and a sample was taken (100 μl). Serial dilutions of each sample were plated on LB agar with and without tetracycline (25 μg ml^–1^). These plates were incubated for 24 h at 27°C, then Colony Forming Units (CFU) were counted. Plates containing tetracycline were compared to those without to ensure there was no more than 10% difference in CFU number. A visual examination for fluorescence under the microscope was also carried out at each 24-hour timepoint. To facilitate a comparison between transformed and non-transformed bacteria, the growth of *P. fluorescens* SBW25 and *P. fluorescens* SBW25 E1433 was measured in LB and a rich defined RD-MOPS media using a micro plate reader (Multiskan Go, Thermo Scientific, United States). Measurements of optical density at 600 nm (OD_600_) were taken every half hour. Bacteria were grown at 21 and 27°C with intermittent shaking. The emission spectrum of the transformed isolate was measured using a plate reader (Varioskan – Lux, Thermo Scientific, United States) with an excitation wavelength of 485 nm and emission range of 510–600 nm.

### Bacterial Growth Conditions and Root Inoculation

*Pseudomonas fluorescens* SBW25 E1433 was removed from storage in 20% glycerol at −80°C, streaked on LB agar plates containing tetracycline (25 μg ml^–1^) and incubated at 27°C for 24 h. A single colony was selected and cultured in liquid LB containing tetracycline (25 μg ml^–1^) for 24 h at 27°C with shaking (200 rpm). A 1:100 sub-culture was then transferred into a rich defined RD-MOPS media containing tetracycline (25 μg ml^–1^). This was incubated for a further 24 h at 18°C with shaking (200 rpm). Bacterial suspensions for inoculation of roots were prepared by diluting this liquid culture to an OD_600_ of 0.02 in 0.5 × MS media. This corresponded to an approximate bacterial density of 2 × 10^7^ CFU ml^–1^. For each treatment, an initial measurement of bacterial density was obtained (CFU ml^–1^) by serial dilution and plating on Kings-B agar (Sigma Aldrich 60786) containing tetracycline (25 μg ml^–1^). Kings-B agar enables the efficient counting of *P. fluorescens* as it encourages the production of fluorescent compounds. All inoculations were carried out at the same point in the light cycle of the growth-cabinet. Prior to inoculation, the 0.5 × MS was removed from microcosm wells using a Pasteur pipette. Approximately 1 ml of either bacterial suspension, or a negative control of 0.5 × MS, was then used to fill the well. Microcosms were returned to the growth-cabinet to await sampling or further treatment.

### Root Sampling and Bacterial Counts

Bacterial colonization density was determined based on CFU counts. At the relevant timepoint for each experiment, plants were removed from microcosms. Each plant was dip washed three times in sterile Phosphate Buffer Saline (PBS). The phyllosphere, the region from the base of the hypocotyl upward, was removed using an ethanol-sterilized razor blade and discarded. Roots were weighed in 1.5 ml sample tubes using a (Ohaus PA214) scale, then homogenized aseptically using a micro-pestle in the sample tube, in 100 μl of PBS. Serial dilutions of each sample were plated on Kings-B agar containing tetracycline (25 μg ml^–1^) and incubated at 27°C for 24 h prior to obtaining a CFU count.

### Analysis of Bacterial Internalization

An assay was carried out to assess the influence of internalization on rhizoplane colonization density. Microcosms containing plants were inoculated as described above. Microcosms were sampled at 2, 24, 48, 72, and 96 h post inoculation. As above, roots were removed from microcosms and separated from the phyllosphere. Roots were then surface sterilized by placing them in 0.03% w/v sodium hypochlorite for 3 min at room temperature with gentle shaking. Imprints were made by placing the roots on Kings-B agar containing tetracycline (25 μg ml^–1^) for ∼ 30 s, followed by incubation at 27°C for 24 h, to assess the effectiveness of the surface sterilization protocol. Internalized bacteria were quantified by CFU counts as above. A minimum of five plants were collected for each timepoint, along with an equal number of non-inoculated negative control plants.

### Analysis of Bacterial Numbers on the Root Surface

**FIGURE 2:**
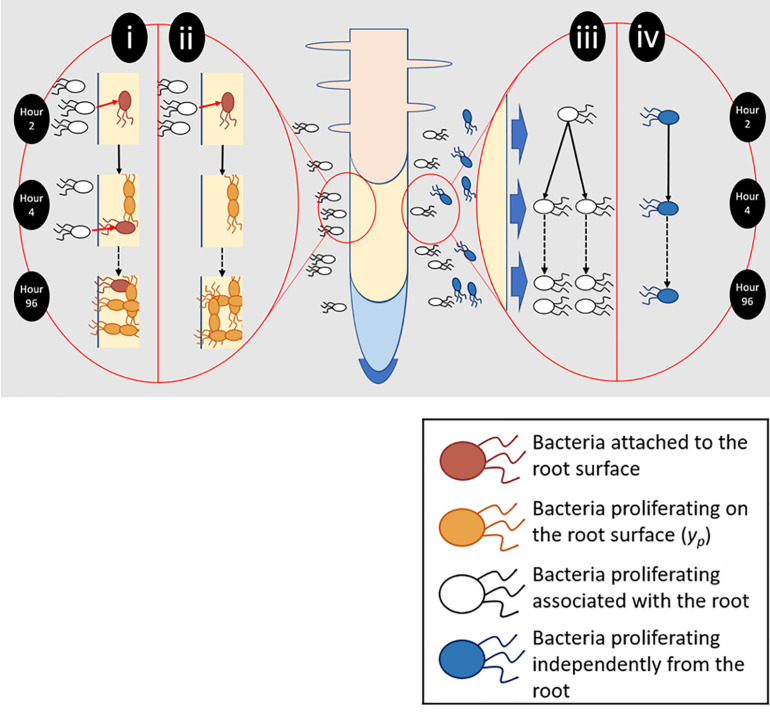
Different aspects of root surface colonization were experimentally isolated and quantified. (i) Total bacterial numbers on the root surface (y_c_) were the result of bacterial attachment (pink) and proliferation (orange). (ii) To quantify proliferation on the root surface (y_p_), plants were transferred to sterile microcosms 2 h after inoculation. Sterile microcosms contained no bacteria in suspension (white) and as a result attachment could not occur. (iii) Bacteria proliferating in the presence of the root and root exudate were quantified. (iv) The ability of bacteria to proliferate in the absence of any root input was also determined.

To quantify total colonization density (*y*_c_), which results from both attachment and proliferation on the root surface, bacterial counts were obtained for entire root systems. Counts were carried out at 2, 18, 24, 48, 54, 72, and 96 h post inoculation, over six runs. Further sampling was carried out every 2 h between 18 and 54 h. For each timepoint, a minimum of five inoculated and non-inoculated (control) microcosms were sampled. To quantify the contribution of bacterial proliferation on the root surface to total colonization density (*y*_p_, Figure 2), plants were inoculated, then gently removed from their chambers 2 h post inoculation. Root systems were rinsed in PBS to remove unattached bacteria. Plants were then placed in fresh, sterile, microcosm chambers. The first set of samples was taken during transferal – 2 h post inoculation. Following this, microcosms were sealed and returned to the growth chamber prior to sampling at 24, 48, 72, and 96 h post inoculation. This experiment was repeated three times with a minimum of five inoculated microcosms per timepoint, along with an equal number of non-inoculated negative controls.

### Analysis of Bacterial Proliferation Surrounding the Root

The ability of *P. fluorescens* SBW25 E1433 to proliferate in the medium surrounding the root was investigated by inoculation of a group of five chambers containing a plant, as well as a control group with no plants, as above (Figure 2). These were placed within the plant growth chamber. Sampling was carried out at 2, 24, 48, 72, and 96 h post inoculation with further sampling every 2 h between 2 and 24 h. At each timepoint, 100 μl of growth medium was taken from a minimum of five chambers from both groups. CFU counts were established based on plating of serial dilutions on Kings-B agar containing tetracycline (25 μg ml^–1^), incubated at 27°C for 24 h.

### Analysis of Bacterial Proliferation in Root Exudate

To study the ability of *P. fluorescens* SBW25 E1433 to proliferate in the exudates of roots grown in hydroponic, and potentially hypoxic conditions, exudates were collected from lettuce plants grown for 8 days in the microcosm system. During the 8 days, plant grew without replenishment of nutrient solution. The liquid solution from 30 microcosms was collected and pooled. Although it was assumed that non-inoculated microcosm chambers remained sterile, exudates were sterilized using a 0.45 μm filter (fisher scientific 10619672). Filtered exudate solution was plated on non-selective LB agar to test for contamination and found to be negative. Benedict’s reagent (Sigma Aldrich 11945) was used for quantification of reducing sugars. Exudate solution was stored at −80°C between experiments. Bacterial growth was quantified in microcosms containing no plants. Liquid cultures of *P. fluorescens* SBW25 E1433 in rich defined RD-MOPS media containing 25 μg ml^–1^ tetracycline were prepared as described above. Cultures were diluted to an OD_600_ of 0.02 in either 0.5 × MS or root exudate solution. 1 ml of either suspension was pipetted into eight microcosm chambers containing no plants. Each chamber corresponded to the exudate collected from a single plant, after pooling to control for plant-plant variation. An equal number of negative controls containing no bacteria were prepared. Microcosms were placed in the plant growth chamber. At 2, 24, 48, 72, 144, and 168 h post inoculation, 100 μl of solution was taken from each chamber. CFU counts were established based on plating of serial dilutions on Kings-B agar, containing tetracycline (25 μg ml^–1^).

### Models for Microbial Growth

Bacterial density on root surfaces (*y*, g^–1^) was determined based on CFU counts and root weight (*Wt, g*). These were normalized based on bacterial density in the inoculant (*CFU*^*o*^) to account for variations in initial conditions,

(1) $$y=\frac{C\,F\,U}{C\,F\,U^{0}\,W\,t}.$$

Data relating to the proliferation of bacteria in the medium surrounding the root, in the absence of the root, and in root exudate were expressed as Log_10_(CFU ml^–1^).

Various classical models of microbial growth were tested on the data. These included the Logistic (Tsoularis and Wallace, 2002), Gompertz (Gibson et al., 1988), Baranyi (Baranyi and Roberts, 1994) and Richards models (Richards, 1959). Model selection, based on the lowest Akaike Information Criterion (AIC) value, led to the use of the Gompertz function for the proliferation of unattached bacteria in the presence of the root,

(2) $$y=K^{*}e{(}^{\left(l\,o\,g\,\text{y0}\,/\,\text{K}\right)}e^{-\mu \,t\,i\,m\,e}).$$

A separate model, representing logistic decline, was used for bacteria in the absence of a root or root exudate,

(3) $$y=a+b\,\left(1-e^{\left(\text{-c}\,/\,\text{time}\right)}\right).$$

The logistic model (Eqs. 4, 5) was the best fit for total increase of microbial density on the root surface (*y*_c_), proliferation on the root surface (*y*_p_) and proliferation in root exudate data. The logistic equation defines the change of bacterial density (*y*) as a function of the intrinsic growth rate (*μ*), and the carrying capacity of the medium (*K*).

(4) $$\frac{d\,y}{d\,t}=\mu \,y\,\left(\frac{K-y}{K}\right).$$

The solutions of Eq. 4 are of the form

(5) $$y=\frac{K\,y_{0}}{y_{0}+\left(K-y_{0}\right)\,e^{-\mu \,t}},$$

with *y*_*0*_ the initial bacterial density. The equation is fitted on experimental data to estimate growth parameter values (*y*_0_, *K*, μ) from each experiment. Because the carrying capacity of the root is an intrinsic property of plant roots, it is assumed to be constant across all conditions will be considered constant in the system.

### Measurement of Attachment and Time for the Recruitment of Bacteria

To determine the relative contribution of attachment and proliferation on overall bacterial density, bacterial density was monitored from two distinct experiments. The bacterial density due to proliferation (*y*_p_) is defined as the bacterial density on the rhizoplane that result from inoculation at the start of the experiment before transferal to sterile microcosms. The total density due to bacterial colonization (*y*_c_) is defined as the bacterial density on the rhizoplane resulting from both proliferation and attachment of bacteria present in the medium. *y*_p_ and *y*_c_ were both measured experimentally (Figure 2). Because the attachment rate *R*_a_ (g^–1^ h^–1^) cannot be measured directly, it must be derived from the difference between the rate of total colonization *R*_c_ (g^–1^ h^–1^) and rate of proliferation on the root surface (*R*_*p*_):*R*_c_ = *R*_*p*_ + *R*_*a*_. The rate of total colonization *R*_c_ (g^–1^ h^–1^) is obtained by differentiation of Eq. 5,

(6) $$R_{c}=\frac{K_{c}\,\left({\text{K}}_{\text{c}}\,/\,y_{c}^{0}-1\right)\,e^{-\mu_{c}}}{{\left[1+\left({\text{K}}_{\text{c}}\,/\,y_{c}^{0}-1\right)\,e^{-\mu_{c}\,t}\right]}^{2}}.$$

*R*_c_ represents the combination of attachment to and proliferation on the root surface. Proliferation on the root surface depends on the density of bacteria on the root as described in Eq. 4. Therefore, in the second step, the contribution of proliferation to colonization rate was determined. This was as a function of *y*_c_ at time *t*, which, according to Eq. 4 is,

(7) $$R_{p}=\mu_{p}\,y_{c}\,\left(\frac{K_{c}-y_{c}}{K_{c}}\right).$$

Here *y*_c_ is the bacterial density determined from Eq. 4 fitted on experimental data for total root colonization density. Finally, the rate of attachment (*R*_a_) is defined as the difference between total colonization rate and proliferation rate,

(8) $$R_{a}=R_{c}-R_{p}=\frac{K_{c}\,\left({\text{K}}_{\text{c}}\,/\,y_{c}^{0}-1\right)\,e^{-\mu_{c}}}{{\left[1+\left({\text{K}}_{\text{c}}\,/\,y_{c}^{0}-1\right)\,e^{-\mu_{c}\,t}\right]}^{2}}-\mu_{p}\,y_{c}\,\left(\frac{K_{c}-y_{c}}{K_{c}}\right)$$

The attachment rate can therefore be expressed as a function of the total colonization density *y*_c_ using the proliferation coefficientμ_p_ (Table 1).

**TABLE 1:** Model variables and parameters.

***Wt***	**Root weight (g)**
*CFU*^0^	Bacterial density of inoculant (ml^–1^)
*y*	Bacterial density on root surfaces (g^–1^)
*K*	Carrying capacity (g^–1^)
*y*^0^	Bacterial density on root surfaces at *t* = 0 (g^–1^)
μ	Intrinsic growth rate
*y*_c_	Total colonization density (g^–1^)
*K*_c_	Root surface carrying capacity (g^–1^)
$y_{\text{c}}^{0}$	Bacterial density on root surfaces at *t* = 0 for total colonization (g^–1^)
μ_c_	Intrinsic growth rate for total colonization (g^–1^ h^–1^)
*y*_p_	Colonization density in the absence of attachment (g^–1^)
$y_{\text{p}}^{0}$	Bacterial density on root surfaces at *t* = 0 in the absence of attachment (g^–1^)
μ_p_	Intrinsic growth rate in the absence of attachment (g^–1^ h^–1^)
*R*_c_	Rate of total colonization (g^–1^ h^–1^)
*R*_p_	Rate of proliferation (g^–1^ h^–1^)
*R*_a_	Rate of attachment (g^–1^ h^–1^)
*p*(*t*)	Contribution of attachment at t to the total colonization of the rhizoplane at *t* = 96 h

To characterize the role of timing in the success of a microbe colonizing the root surface, we quantified the relative contribution of attachment at any given time (*t*) to the total colonization of the rhizoplane at the end of the experiment. This was calculated as the proportion (*p*) of the final quantity of bacteria that originate from those attached at time *t*,

(9) $$p\,\left(t\right)=\frac{R_{a}\,\left(t\right)}{K_{c}}\,\int_{t}^{96}\mu_{p}\,y_{c}\,\left(\frac{K_{c}-y_{c}}{K_{c}}\right)\,d\,t.$$

### Software and Statistical Analyses

Modeling and data analysis were carried out using R (R Development Core Team, 2018). Individual replicates of each treatment type were pooled and analyzed together. Models were fit to each data set using the R package, growthrates (Petzoldt, 2019). Time was given by hour for all data sets. Error for selected models was calculated by bootstrapping with 1000 replicates. To ensure bias was not introduced by the use of parametric forms in calculation of attachment parameters, the same calculations were also done with non-parametric cubic spline fitting on both total colonization density and proliferation data sets. Rate of change of total colonization density (*R*_c_) and proliferation on the root surface (*R*_p_) were calculated based on the finite difference approximation of the derivative of the splines. The relationship between root weight and Log_10_(CFU ml^–1^) per root, for total colonization density and proliferation data, was investigated by preforming a linear regression. This was carried out for data at 96 h. Source code can be downloaded at https://github.com/DaireCarroll2019/Root-Attachment-Modeling.

## Results

### Root Exudation and Bacterial Proliferation on or Near the Rhizoplane Are the Main Factors Contributing to Colonization

Imprints of lettuce seeds on non-selective LB agar were found to be clean, indicating that surface sterilization was successful in removing bacteria from the surface of the seed. Plates containing homogenized roots from non-inoculated, negative control chambers for subsequent experiments were also found to be clean. This suggested that non-inoculated microcosms remained free of contamination and *P. fluorescens* SBW25 E1433 did not compete with other microorganisms during these experiments.

To identify the factors influencing the colonization of lettuce roots by *P. fluorescens* SBW25 E1433, plants were grown in a microcosm set up (Figure 1) enabling the quantification of bacterial numbers on the root surface. Roots were either grown continuously in one microcosm, enabling both attachment and proliferation on the root surface, or transferred to sterile microcosms 2 h post-inoculation enabling a quantification of proliferation in the absence of attachment (Figure 2).

**FIGURE 3:**
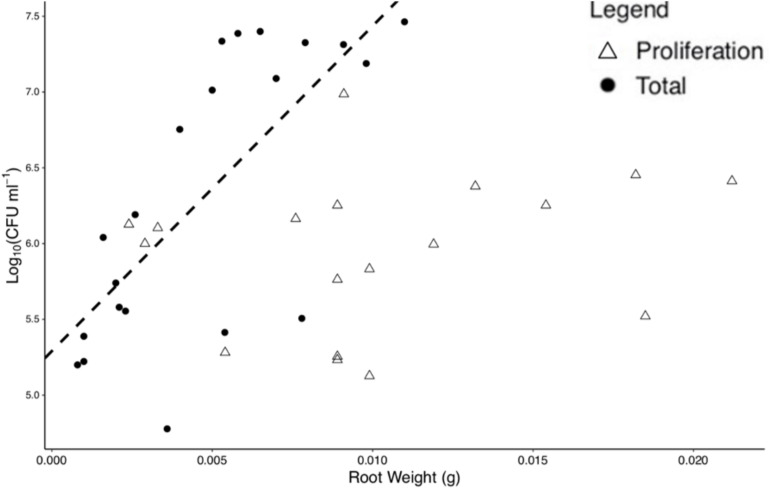
Bacterial numbers on the rhizoplane and root weight are positively correlated for total colonization at 96 h. Each point represents an individual destructive measurement of root weight and Log_10_(CFU) on the root surface. A significant linear relationship was found for total colonization density at 96 h but not for proliferation data. The dashed line represents the linear model for total colonization (slope = 214.551 Log_10_(CFU) ml^– 1^, intercept = 5.291 Log_10_(CFU) ml^– 1^).

For total colonization, root weight was found to be positively correlated with bacterial numbers at *T* = 96 h (*p* < 0.01, *N* = 20, *DF* = 19, *SE* ≤ 0.01, *R*^2^ = 0.51), with a slope of 214.55 Log_10_(CFU) ml^–1^ and an intercept of 5.29 Log_10_(CFU) ml^–1^, based on a linear regression (Figure 3). No significant correlation was found for root weight and bacterial numbers for proliferation (*p* = 0.43, *N* = 17, *DF* = 16, *SE* = 0.01, *R*^2^ = 0.04) alone. Root weight and total CFU count were used for the determination of normalized colonization density (*y*, Eq. 1) to control for variation in plant size across replicates.

Next, the presence of bacteria internalized within root tissue was quantified. All root imprints were found to be clean, indicating that surface sterilization was successful in removing bacteria from the rhizoplane. We found that internalization was limited to <0.2% of mean total colonization density (g^–1^) in the root tissue at the final timepoint (*T* = 96 h). Internalization was therefore considered insignificant as a distinct contributing factor and so not included in further analyses.

**FIGURE 4:**
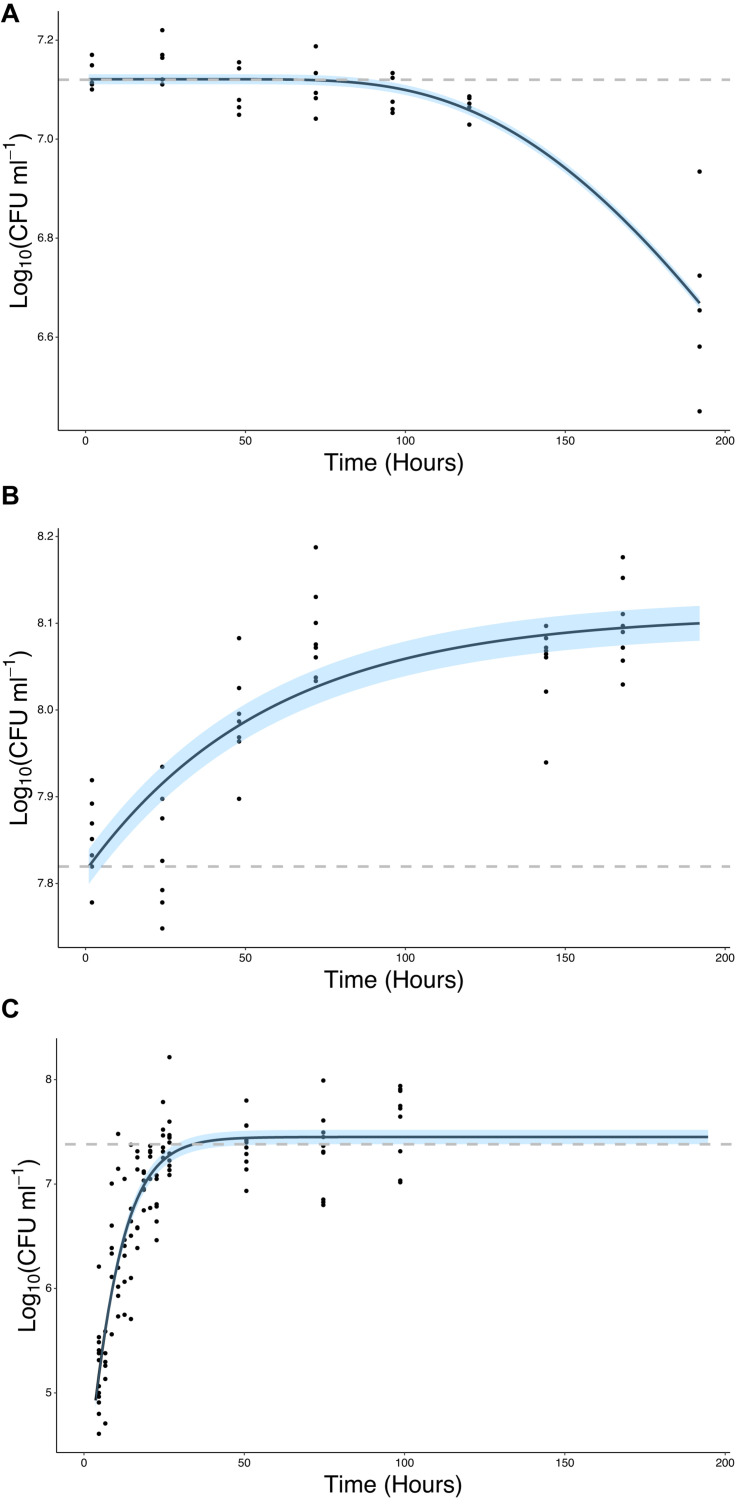
Bacteria proliferate in the presence of the root or root exudate, but not in the absence of any root input. **(A)** The change in Log_10_(CFU) of *P. fluorescens* SBW25 E1433 in a microcosm system over time in the absence of a plant root or root exudate. Here bacterial numbers in suspension were quantified in microcosm systems containing 1/2 MS plant growth media and no plant (Figure 2 iii). **(B)** The change in Log_10_(CFU) of *P. fluorescens* SBW25 E1433 in a microcosm system over time in the absence of a plant root but presence of root exudate. Here bacterial numbers in suspension were quantified in microcosm systems filled with exudate from lettuce roots (Figure 2 iii). **(C)** The change in Log_10_(CFU) of *P. fluorescens* SBW25 E1433 in a microcosm system over time in the presence of a plant root. Here bacterial numbers in suspension were quantified in microcosm systems containing a plant (Figure 2 iv). Black lines represent the relevant fitted model (Table 1). Gray dashed lines represent the average initial value for Log_10_(CFU) in the original inoculant across different replicates of the same treatment. Shaded regions represent bootstrap errors.

The ability of bacteria to grow in the presence of root exudates, produced in hydroponic conditions, was also quantified. In the absence of a root or any other plant input, the bacterial count remained constant, with a mean value of 7.12 Log_10_(CFU ml^–1^) until *T* = 96 h at which point it began to decline, ending up at a mean value of 6.67 Log_10_(CFU ml^–1^) at *T* = 192 h (Figure 4A). In contrast, bacterial density increased in the presence of root exudates. Bacterial density increased up to a mean maximum value of 8.09 Log_10_(CFU ml^–1^) which was reached at *T* = 72 h (Figure 4B), rising from a mean of 7.85 Log_10_(CFU ml^–1^) at *T* = 2 h. Sterile root exudates (pooled) were found to have a reducing sugars content of 0.25% w/v. In the presence of a root, bacterial density increased up to a mean maximum value of 7.41 Log_10_(CFU ml^–1^), reached at *T* = 24 h, rising from a mean of 5.22 Log_10_(CFU ml^–1^) at *T* = 2 h. The best fit for growth in the absence of a root or root exudate was obtained with the logistic decline model (Eq. 3, AIC = −76.92, *r*^2^ = 0.82, *SE* = 0.08, *N* = 33, Figure 4B and Table 2). The best fit for growth in root exudate obtained with the logistic model (Eq. 4, AIC = −16, *r*^2^ = 0.87, *SE* = 0.68, *N* = 46, Figure 4C and Table 2). The best fit for growth in the presence of a root was obtained with the Gompertz model (Eq. 2, AIC = 123, *r*^2^ = 0.78, *SE* = 0.41, *N* = 112, Figure 4C and Table 2).

**TABLE 2:** Models selected based on AIC value for each data set along with model parameters.

**Data Set**	**Selected Model**	**Bootstrap (1000) Error**	**Parameters**	***P* value**
Total Colonization	Logistic	0.54	*K* = 8.855974*y*0 = 0.007333μ = 0.184677	*p* < 0.001*p* = 0.559*p* < 0.001
Proliferation on root surface	Logistic	0.82	*K* = 9.04029218*y*0 = 0.02508862μ = 0.09949397	*p* = 0.001*p* = 0.6409*p* = 0.0117
Proliferation in root presence	Gompertz	0.07	*K* = 7.4503415*y*0 = 4.6670427μ = 0.1240495	*p* < 0.001*p* < 0.001*p* < 0.001
Proliferation in root absence	Logistic decline	0.01	*a* = −5.151*b* = 12.272*c* = 633.798	*p* = 0.66632*p* = 0.30719*p* = 0.00181
Proliferation in exudate	Logistic	0.02	*K* = 8.109634*y*0 = 7.814389μ = 0.017965	*p* < 0.001*p* < 0.001*p* = 0.177

### Attachment and Proliferation Contribute Differently to Rhizoplane Colonization

**FIGURE 5:**
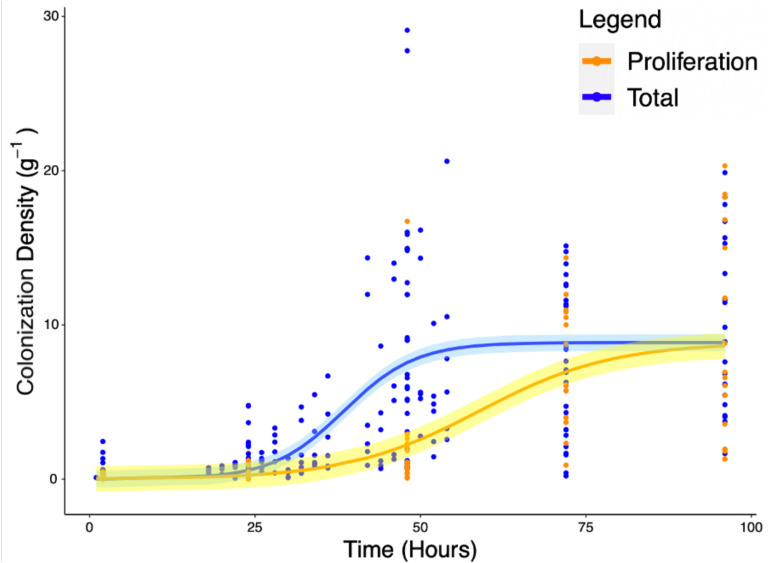
Increase of total root surface colonization density (blue) as well as increase due to proliferation (orange) were modeled using the logistic equation. The accumulation of *P. fluorescens* SBW25 E1433 on root surfaces over time is shown with root surface colonization density represented by CFU normalized for original inoculant and root weight (Eq. 4). Each point represents a destructive measurement of CFU on the root surface of an individual plant. Lines represent fitted logistic models. The total values, shown in blue, represent the bacteria present on the root due to both attachment and proliferation (total colonization density). The proliferation values, shown in orange, represent bacteria proliferating on the root in the absence of attachment beyond 2 h. Shaded regions represent bootstrap errors.

Experiments carried out to quantify the density of bacteria on the root surface (*y*_c_) showed that there is consistent increase in microbial density with time as part of the colonization process. Total colonization of root surfaces reached a mean plateau of 9.97 CFU g^–1^ at *T* = 72 h, rising from a mean of 0.4 CFU g^–1^ at *T* = 2 h. A similar form of growth was observed in experiments carried out to quantify the density of bacteria (*y*_p_) in the root proliferation experiment. When roots were inoculated at the start of the experiment and subsequently transferred to sterile microcosms, a consistent increase in bacterial density was observed on the rhizoplane. Although the extent of cell density increase declined marginally, it continued to increase up to *T* = 96 h with a mean value of 8.78 CFU g^–1^, rising from a mean of 0.13 CFU g^–1^ at *T* = 2 h. The density of bacteria observed on the root remained variable between sample replicates, despite the normalization, showing that there was biological variation between bacterial populations and plants. Fitting of classic bacterial growth models on experimental data provided useful parameters for understanding the process of colonization (Table 1). The best fit for colonization and proliferation were obtained with the logistic model with respective fit parameters of AIC = 1286, *r*^2^ = 0.42, *SE* = 0.56, *N* = 223) and AIC = 498, *r*^2^ = 0.48, *SE* = 3.32, *N* = 88 (Figure 5 and Table 2).

### Mathematical Modeling Allows Decoupling of Proliferation From Attachment Rate on the Rhizoplane

**FIGURE 6:**
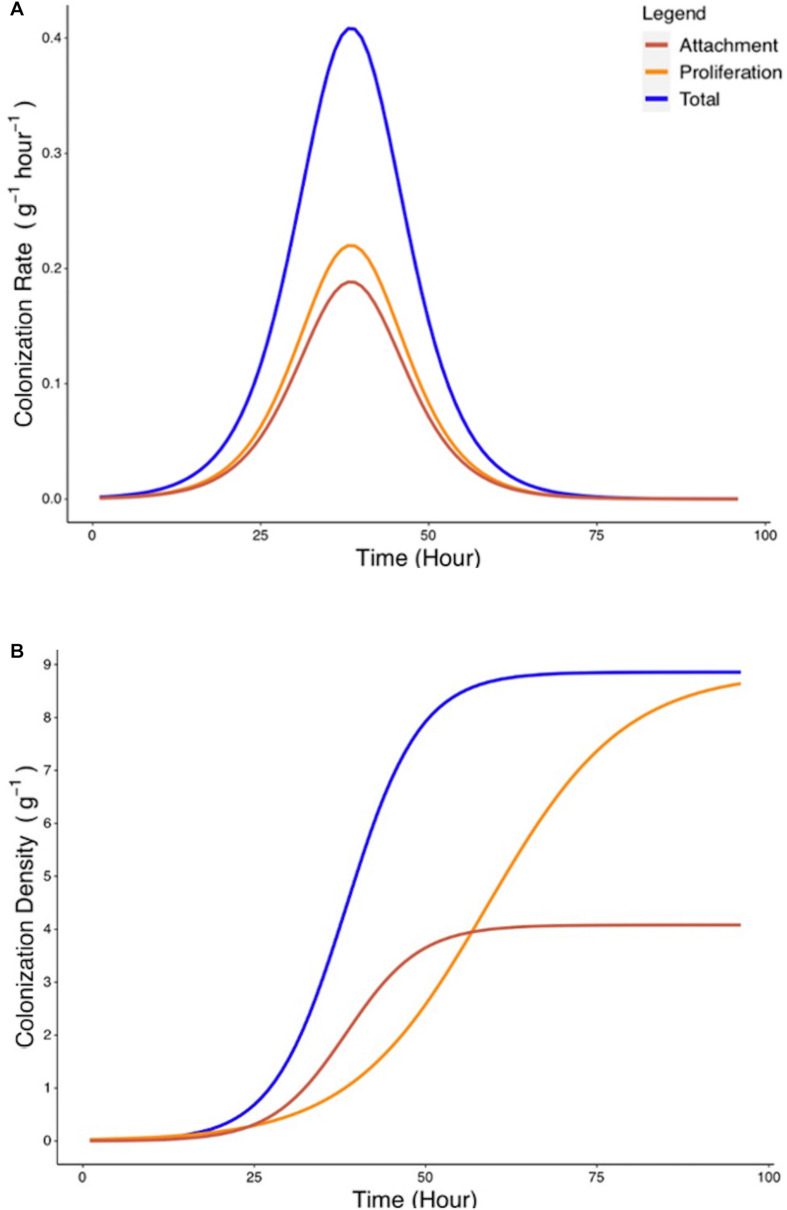
Estimated rate of bacterial attachment to the rhizoplane. **(A)** The rates of total colonization (R_c_, blue) and proliferation (R_p_, yellow) were calculated based on Eqs. 6, 7. The rate of attachment (R_a_, red) was calculated based on Eq. 8. **(B)** Colonization density due to attachment was estimated by integration of Eq. 8.

Since attachment rate cannot be measured directly from experimental methods, a mathematical framework was developed (Eqs. 6–9) to estimate such parameters from experimental growth curves *y*_c_ and *y*_p_. Estimation of the attachment parameters was achieved by the following steps. First, growth curves *y*_c_ and *y*_p_ were used to determine the experimental colonization and proliferation rates. In a second step, the proliferation rate was expressed as a function of the density of bacterial colonization. The attachment rate was subsequently calculated as the difference between total colonization rate and the proliferation rate during the total colonization experiment (Figure 6A). Finally, the total quantity of bacteria present on the root surface due to recruitment from the surrounding media can be calculated from the attachment rate by integration (Figure 6B). Attachment rate estimated using this approach exhibited similar kinetics to microbial colonization. A peak of 0.18 g^–1^ h^–1^ in attachment rate is achieved at *T* = 38 h. This indicates that the level of colonization of the root affects the attachment rate of bacteria. Attachment rates calculated based on cubic splines did not show disagreement from those generated by treatment of parametric models. This suggests that no bias was introduced by the choice of growth models.

### Factors Contributing to Attachment and Colonization

**FIGURE 7:**
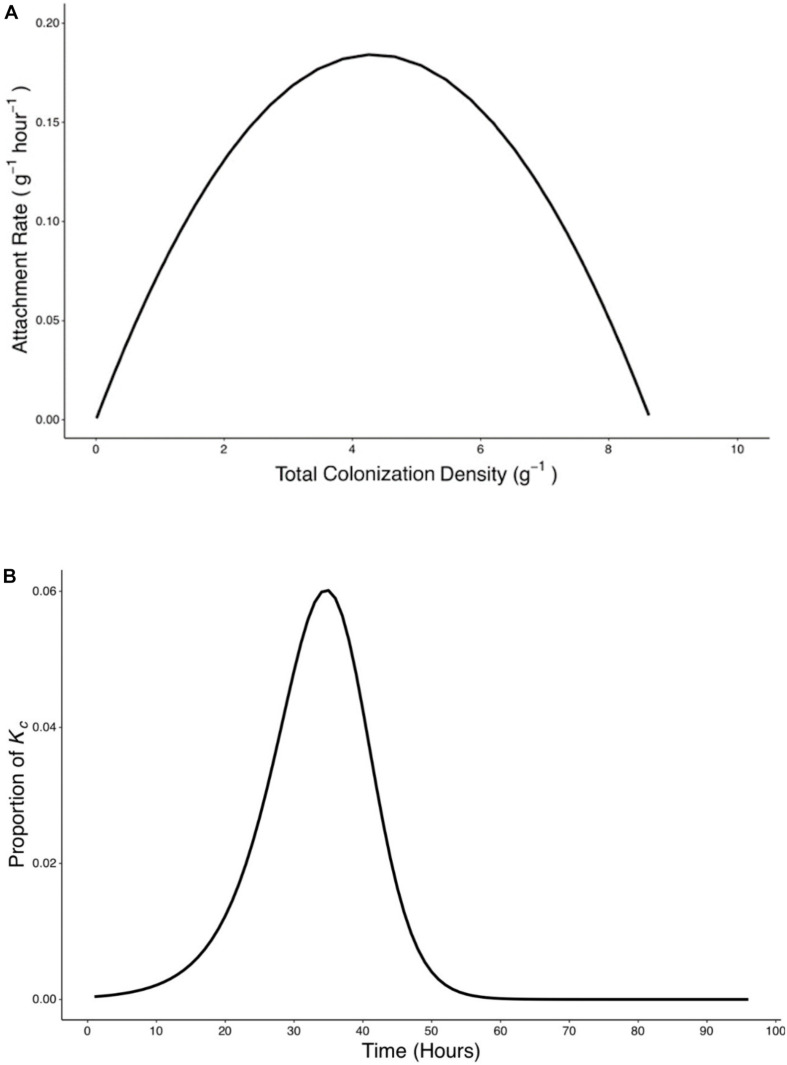
Rate of attachment (*R*_*a*_) expressed as a function of total colonization density (*y*_*c*_) and the relative contribution of attachment to final colonization density was established. **(A)** The relationship between rate of attachment and total colonization rate can be expressed as a quadratic function (Ra=-1.19⁢x⁢ 10-11+8.52⁢x⁢ 10-2⁢yc-9.98⁢x⁢ 10-3⁢yc2). **(B)** The proportion of K_c_ reached at 96 h by bacteria which attach at any time p(t) was calculated based on Eq. 9.

Attachment rate *R*_a_ (g^–1^ h^–1^) was found to vary over time, rising from a starting value of 7.5 × 10^–4^ g^–1^ h^–1^ to a peak value of 0.188 g^–1^ h^–1^ at *T* = 38 h before declining to a value of 1.82 × 10^–5^ g^–1^ h^–1^ at *T* = 96 h (Figure 6B). To investigate the influence of total colonization density on attachment rate, *R*_a_ was expressed as a function of *y*_c_, the total colonization density (g^–1^) on the root surface. It was found that this relationship could be expressed as a quadratic equation (Figure 7A),

(10) Ra=-1.19⁢x⁢ 10-11+8.52⁢x⁢ 10-2⁢yc-9.98⁢x⁢ 10-3⁢yc2

A peak of 0.19 g^–1^ h^–1^ in attachment rate was seen when total colonization density was at 4.26 g^–1^. This corresponded to the attachment and colonization values at 38 h post inoculation (Figure 7A). Results also show the timing of attachment influenced the extent of successful colonization of the microbe on the root. Bacteria proliferation rate was used to calculate the contribution of attached bacteria at time *t* to the total density of bacteria at the end of the experiments (Figure 7B).

## Discussion

The experimental approaches proposed in our study are in line with a long series of past studies for measurement of root colonization (Hansen et al., 1997; Schmidt et al., 2018) and assessment of attachment of bacteria to roots (Mills and Bauer, 1985; Albareda et al., 2006). Destructive quantification of root colonization is generally carried out at a single timepoint or at very coarse time intervals (Unge and Jansson, 2001; Schmidt et al., 2018). Unattached bacteria can be removed by washing, with numbers of attached bacteria subsequently being determined through either plating or imaging. Such assays are commonly used in plant and bacterial sciences, however, as the destruction of the sample is required, they lack the temporal resolution necessary to map out the dynamic process of colonization. Our experimental system shares the same limitations, but significant effort was put into quantifying colonization at dense time intervals during the early stages of colonization for the data to capture the precise kinetics of attachment on the root.

Efficient colonization is a key component of plant growth promoting bacterial activity (Chin-A-Woeng et al., 2000; Kamilova et al., 2005). As a result, quantification of colonization is important for assessing plant growth promoting bacterial strains (Mendis et al., 2018), although it is often overlooked (Cipriano et al., 2016; Kour et al., 2019). Destructive quantification methods, similar to that used in our experimental system, can be used to assess colonization of roots by plant growth promoting bacteria (Bach et al., 2016; Hsu and Micallef, 2017).

We observed similar bacterial colonization levels to those reported previous studies. Noirot-Gros et al. (2018) reported 5 × 10^7^ CFU g^–1^ of root after 5 weeks, on aspen (*Populus tremula*). Unge and Jansson (2001) studied the colonization of wheat roots by *P. fluorescens* isolate SBW25, a plant growth promoting bacterial isolate, at 6 days post inoculation and reported root colonization values between 1.15 × 10^8^ and 4.29 × 10^8^ CFU g^–1^ of root. We reported slightly lower mean colonization density 9.1 × 10^6^ CFU g^–1^ of root at *T* = 96 h. The lower values we report are unlikely to be the result of shorter experimental times, as the logistic growth model predicted that carrying capacity would be reached during our experimental timeframe (Figure 5). Instead, differences in colonization levels are likely due to root maturity, plant species and quantification method. Studies based on short exposure of the root to bacteria have had limited scope because colonization rate is affected by a range of factors (Massalha et al., 2017; Schmidt et al., 2018).

Colonization assays are powerful because of their simplicity and ability to study large numbers of samples in one experiment. However, using such screens for characterization of attachment or proliferation rates is more difficult. They cannot distinguish attachment from proliferation on the root meaning individual rate parameters cannot be obtained directly. Attachment rate is a particularly difficult parameter to measure since direct observation and tracking of single bacterial cells is rarely achievable in the root environment. A typical approach is to quantify attachment by viable cell counts after a short period of exposure to bacteria (Shimshick and Hebert, 1979; Albareda et al., 2006), during which time increase in bacterial density due to proliferation is limited. Mills and Bauer (1985) preformed a quantification of the attachment of *Rhizobium trifolii* to white clover (*Trifolium repens*), using root sonication and enumeration to quantify attached cells. Variations of these approaches have been tested on a range of bacteria and plant species (Albareda et al., 2006).

In this study, we have addressed the limitations of colonization and attachment assays using both data with high temporal resolution and a suitable mathematical framework linking colonization and proliferation rates. This allowed the calculation of system parameters unobtainable using traditional methods. We observed a notable time lag required for permanent attachment (approximately 24 h). This was not detected in previous studies, probably due to differences in method of extraction that counted non-permanent attachment of bacteria. Our method also allows resolution of time variations in colonization rate not previously available. Attachment rate varies with time, due to the changing density of both attached and free moving bacteria, as well as transient and heterogeneous adherence factor gene expression profiles of bacteria. This was established by the early work of Shimshick and Hebert (1979), who proposed a dynamic model of attachment on roots based on adsorption-desorption theory. However, the scope of the study by Shimshick and Hebert (1979) is limited because the model did not consider the proliferation of bacteria on the root surface itself, which we showed is not negligible (Figure 4).

### Application of Mathematical Framework for Estimation of Attachment Rate During Bacterial Establishment

Our mathematical estimations of bacterial attachment rates have broad applicability. They rely on standard colonization assays commonly used in laboratories. The method does not require sophisticated live observations of bacteria, and calculations for estimation of growth and attachment coefficients are simple. The method also provides temporally resolved measurements of attachment rate, which is extremely time consuming in dedicated attachment assays. Currently, limitations are linked to the simplified experimental system and how quantification of bacterial density is achieved. The experimental system is highly simplified with comparison to rhizosphere development in natural environments. The lack of physical structure in the substrate is most likely a source of bias in the estimation of attachment rate. Reliably recovering and quantifying a bacterial strain in the field using a plating method is difficult, and only culturable bacteria can be studied by plating. More specific molecular methods for quantification of specific strains, or taxa, are available (Mendis et al., 2018). There is evidence from the literature to suggest adequate data could be obtained from more sophisticated experimental system and modern analytical tools. Colonization data could be obtained from roots grown in natural soils by fluorescent *in situ* hybridization (Gamez et al., 2019), sequencing (Mitter et al., 2017) or qPCR (Mendis et al., 2018). Fluorogenic PCR assays, for example, have been used to quantify the presence of non-culturable *Pseudomonas* in natural soils (Lloyd-Jones et al., 2005). Hydroponic solutions can be replaced with transparent soil which has been shown to provide the physical structure of a soil while enabling direct observation of root and bacteria (Downie et al., 2015).

Because colonization assays rely on plating, they are destructive and require large replication numbers. Colonization assays do not provide maps of spatial variations in attachment rate and use of hydroponics neglect the role of transport to the root surface. Such limitation can be remediated to fully exploit the mathematical framework developed here. Modern live-microscopy can overcome the limitations of colonization assays (Downie et al., 2015; Noirot-Gros et al., 2018). For example, Gamez et al. (2019) compared the root colonization patterns of two plant growth promoting bacterial strains, Pseu*domonas fluorescens* Ps006 and *Bacillus amyloliquefaciens* Bs006, on banana. They concluded that *B. amyloliquefaciens* was a faster colonizer. Modern microscopes provide the ability to image large samples in high throughput (Berthet and Maizel, 2016), to grow plants vertically with automated tracking of root tips (von Wangenheim et al., 2017), and simultaneously map the distribution of bacteria around the root (Massalha et al., 2017; Pavlova et al., 2017). Processing of data using artificial intelligence can automate the mapping of bacterial density along the root (Carbone et al., 2017). The ability to track bacteria has drastically improved since the early work of Shimshick and Hebert (1979), for example, observation of single bacterial cell and visualization of their attachment is now routinely achieved with modern microscopes (Duvernoy et al., 2018; Ipina et al., 2019). Mathematical frameworks will be essential to interpret such complex experimental data because they can establish links between attachment rates, root growth, bacterial proliferation, and the complex distribution of bacterial density along the root (Dupuy and Silk, 2016).

### Microbial Establishment on Rhizoplanes

The exact attachment mechanisms of *Pseudomonas fluorescens* isolate SBW25 have not yet been determined. Exploring the dynamics of rhizoplane colonization gives clues as to what might be occurring when bacteria first interact with roots. Based on the results of this study, we can propose various stages of bacterial establishment on root surfaces. In the first step, roots and microbes come into contact. In the case of a hydroponic solution, root exudates diffuse, leading to bacteria detecting the presence of the root and rapidly moving toward it. Secondly, bacteria likely form weak, reversible attachments to the root surface. This establishes a large proportion of the bacterial population in close association with the root. During this stage, rate of proliferation of bacteria surrounding the root increases. This accounts for the low rate of colonization predicted by our model during *T* = 0–24 h. As a third step, strong, irreversible attachment to the root is established. At this stage, the recorded rate of attachment begins to rapidly increase. Attached bacteria proliferate, further increasing colonization rate during *T* = 24–38 h. We predicted that colonizers between *T* = 24–48 h would make the greatest contribution to final root colonization density at capacity, suggesting a dependence of attachment rate on colonization density. Based on our modeling, the involvement of these factors suggests a level of priming activity. Attachment and proliferation rates begin to decrease (*T* = 38 h) before reaching zero in the fourth and final stage (*T* = 38–72 h). At carrying capacity, the rate of new bacteria colonizing the rhizoplane through recruitment and proliferation will be balanced by death, dissociation, and dilution of colonies through root growth. Carrying capacity is the result of limiting factors on bacterial growth. The two most likely limiting factors are space and nutrient availability. The system reaches capacity when the rate of production of new regions, through root growth, is matched by the rate of colonization. The system will be maintained at capacity if root growth rate and colonization rates remain in equilibrium. Longer term, the capacity may also be determined by the rate of nutrient production and the availability of carbon and nitrogen within the rhizosphere has been linked to root size in previous studies (Guyonnet et al., 2018).

## Conclusion

The ability to model rhizoplane colonization is a valuable tool for researchers. Modeling of bacterial interaction with plants is a complex process requiring a solid base in experimental data. Isolating and quantifying aspects of root surface colonization has been shown here to allow the contributions of attachment and proliferation of bacteria root maturity to be estimated, and thus this is an important step in understanding the process of rhizoplane colonization. Our experimental and mathematical frameworks provide a novel method for inferring attachment and proliferation rates during the early period of colonization. This has never previously been possible as these processes are not quantifiable through direct observation. The utilization of plant growth promoting and pathogen suppressing bacteria in agricultural systems will require a solid understanding of the colonization process which has not previously been available. Applications of these novel frameworks include the selection of traits promoting maintenance on the root of beneficial bacteria or limiting the impact of soil borne pathogens. The work presented gives new insight into the interaction between *Pseudomonas fluorescens* isolate SBW25 and Lettuce. It sets the groundwork for more targeted and in-depth studies of rhizoplane colonization, and a more holistic understanding of the interactions between bacteria and plant roots.

## Data Availability Statement

All datasets presented in this study are included in the article/supplementary material.

## Author Contributions

DC carried out the wet lab work and data collection along with design of the microcosm system, carried out the data analysis and modeling with advice from LXD, produced the figures, and wrote the initial manuscript draft and edited with advice from LXD, MLG, and NH. LXD gave advice and assistance in the modeling and downstream data analysis and suggested significant revisions to the text of the manuscript and figures. NH and MLG provided supervision of wet lab experiments and suggested significant revisions to the text of the manuscript and figures. All authors contributed to the article and approved the submitted version.

## Conflict of Interest

The authors declare that the research was conducted in the absence of any commercial or financial relationships that could be construed as a potential conflict of interest.
